# High-power, high-wall-plug-efficiency quantum cascade lasers with high-brightness in continuous wave operation at 3–300μm

**DOI:** 10.1038/s41377-025-01935-6

**Published:** 2025-07-25

**Authors:** Manijeh Razeghi, Yanbo Bai, Feihu Wang

**Affiliations:** 1 Center for Quantum Devices, Department of Electrical Engineering and Computer Science, Northwestern University, Evanston, IL 60208 USA; 2 Quantum Science Center of Guangdong-Hongkong-Macau Greater Bay Area, Shenzhen, 51800, China; 3 Institute for Semiconductor Epitaxy and Devices, Southern University of Science and Technology, Shenzhen, 518055, China

**Keywords:** Quantum cascade lasers, Semiconductor lasers

## Abstract

Quantum cascade lasers (QCLs) are unipolar quantum devices based on inter-sub-band transitions. They break the electron-hole recombination mechanism in traditional semiconductor lasers, overcome the long-lasting bottleneck which is that the emission wavelength of semiconductor laser is completely dependent on the bandgap of semiconductor materials. Therefore, their emission wavelength is able to cover the mid-infrared (mid-IR) range and the “Terahertz gap” that is previously inaccessible by any other semiconductor lasers. After thirty years development, QCLs have become the most promising light source in the mid-IR and THz regime. In this paper, we are going to present the strategies and methodologies to achieve high-power, high-wall-plug-efficiency (WPE) QCLs with high-brightness in room temperature continuous-wave (cw) operation at 3–300 μm. We will also review the recent breakthroughs in QCL community, especially the high-power, high WPE intersubband lasers in room temperature cw operation.

## Introduction

**Fig. 1:**
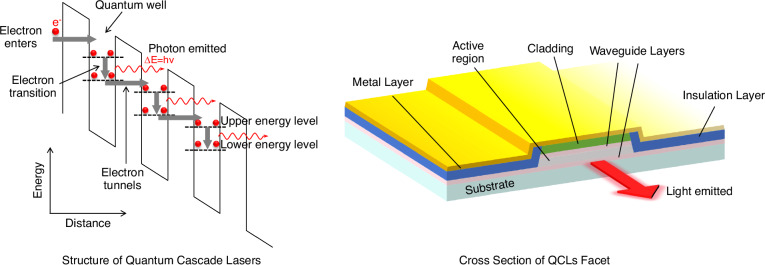
The working principle of quantum cascade laser and its geometry structure. The vertical axis represents energy and the horizontal axis represents the growth direction of materials. The periodically alternating structure at left side represents quantum wells and barriers. The black dashed lines represent artificial energy levels arising from quantum confinement. The black dots presented electrons. The green arrows represent electron injection to upper energy levels. The red wavy lines represent photon emissions originating from electrons transitions from upper energy levels to lower energy levels. After laser transition in a period, electrons will be transported (injected) from the lower energy level of this period to the upper energy level of the next period in the downstream for further transition and photon emission. This process will be repeated in each period and thus considerably increase the gain of lasers

Quantum cascade lasers (QCLs) are semiconductor lasers that emit light in the mid- to far-infrared portion. Unlike traditional interband semiconductor lasers that emit electromagnetic radiation through electron–hole pairs recombination, QCLs are unipolar intersubband lasers, whose lasing action is achieved in an artificial engineered quantum system formed by a repeated stack of semiconductor multiple quantum well heterostructures as shown in Fig. 1. In this figure, the periodically alternating structure represents multiple quantum well heterostructures. The black dots are electrons and the black dashed lines represent artificial energy levels arising from quantum confinement. The red wavy lines represent photon emissions due to electrons transitions from upper energy levels to lower ones. After laser transition in period *i*, electrons will be transported through quantum tunnelling from lower energy level of period *i* to upper energy level of period *i* + 1 in the downstream for further transition. This idea of using intersubband transitions to provide gain was first proposed by R. F. Kazarinov and R. A. Suris in the article “Possibility of amplification of electromagnetic waves in a semiconductor with a superlattice” in 1971^1^, and the first electrical pumped intersubband laser-QCL were demonstrated in 1994^2^, with emission wavelength at ~4 μm, 2340 cm^-12^.

**Fig. 2:**
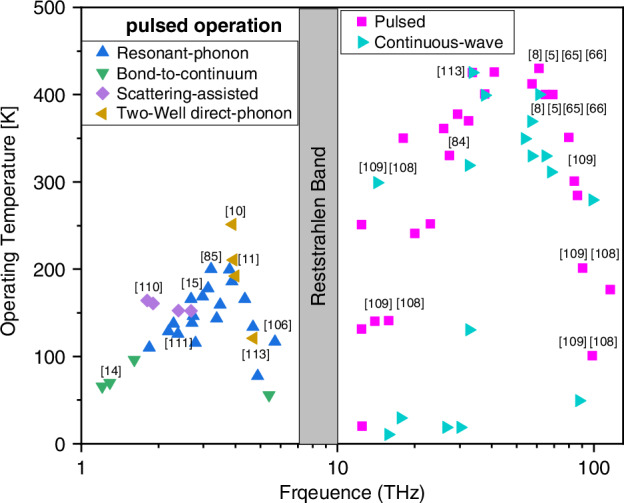
Summarized wavelength coverage of QCLs vs. maximum operation temperature^105–111^. The left side shows the maximum operation temperature of QCLs with different designs in pulse mode operation. The right side shows the maximum temperature performance of mid-IR QCLs in pulse mode and continuous-wave mode operation

As mentioned above, this unipolar semiconductor device was based on inter-subband transitions in quantum wells, instead of inter-band transitions between conduction and valence bands of standard laser diodes^3^. Its emission frequencies and bandwidth could be therefore easily engineered by changing the width of the quantum wells, instead of changing the semiconductor materials. This breakthrough overcame the ‘bandgap slavery’ limitation of the wavelength emission of semiconductor lasers. Since 1994, there have been considerable achievements in the performance of QCLs, including significant increases in output power^4–8^, maximum operating temperature^9–11^ and emission wavelength range^12,13^. Figure 2 shows the QCLs with highest operation temperature at different wavelength. The QCLs with two-well direct-phonon design (brown triangle) have the highest temperature performance up to 261 K at ∼4 THz^10^. The QCLs with bond-to-continuum design (green triangle) have highest temperature performance at longer wavelength^14^. The QCLs with resonant-phonon depopulation design have the highest temperature performance across a wider wavelength range compared with other designs^15^.

In 2002, this concept was extended to the THz range with the first THz semiconductor laser – the THz QCL^13^. This promising THz source is compact, powerful and coherent, permitting a plethora of possible applications from spectroscopy, imaging, telecommunication, biology, medical treatment to fundamental research.

In this paper, we are going to review the development of QCLs and present the strategies and methodologies to achieve high-power, high-WPE intersubband lasers with high-brightness in room temperature continuous wave operation at 3–300 μm, including GaAs-based QCLs and InP-based QCLs.

## QCL Design And Simulation

### Band structure calculation

#### Band structure model

The active medium of QCL consists of several hundred pairs of quantum wells and barriers. The quantum confinement effect to electrons in this quantum system constructs the band structure of QCLs. For QCLs, carrier transport and optical transition take place in the conduction band. Thus, the Ben Daniel-Duke model (one-band effective mass method^16,17^), is often used in QCL modelling. Ignoring the in-plane effective mass dependency in the growth direction, the Ben Daniel-Duke model reduces to the one-dimensional Schrödinger equation:

1 $$\left[-\frac{{\hslash }^{2}}{2}\frac{\partial }{\partial z}\frac{1}{{m}^{* }(z)}\frac{\partial }{\partial z}+{V}_{c}(z)\right]\psi (z)=E\psi (z)$$

where $\hslash$ is the Plank constant, ${m}^{* }$ is the effective mass in the growth direction. ${V}_{c}$ is the conduction band potential profile. And $E$ is the eigen-energy. Generally, the effective mass depends on the material composition and strain. Temperature and doping play minor roles and usually ignorable^18,19^. However, these two parameters play a key role in carrier distribution evaluation. The quantization effective mass is generally different from the in-plane effective mass in strained materials^20^. And m* can be calculated using the method discussed in ref. ^21^, and *E* is the eigenenergy at the minima of band valley. Although Eq. (1) remains an approximation, it is perhaps the most widely adopted method for guiding the design of QCLs.

Nonparabolicity plays an important role in QCL modelling for accurately predict the optical transition energy and injection alignment in band structure design^21,22^. The model above is extended to include the nonparabolicity effect, and hence the effective mass in Eq. (1) is also dependent on the eigenenergy^21^. The quantization and in-plane effective mass read^21,23^: ${m}^{* ,n}(z,E)={m}^{* }(1+{\alpha }^{{\prime} }{E}^{{\prime} })$, and ${m}^{\parallel ,n}(z,E)={m}^{* }(1+\left(2{\alpha }^{{\prime} }+{\beta }^{{\prime} }\right){E}^{{\prime} })$, where ${E}^{{\prime} }=E-V$; ${\alpha }^{{\prime} }$ and ${\beta }^{{\prime} }$ are the nonparabolicity parameters extracted from the 14-**k.p** calculation. For example, in GaAs, ${\beta }^{{\prime} }$ is equal to ${\alpha }^{{\prime} }$ and in InGaAs $2{\alpha }^{{\prime} }+{\beta }^{{\prime} }=1.7{\alpha }^{{\prime} }$ was found experimentally^24^. The correction of the in-plane effective mass is ***z***-dependent. Such dependency is averaged according to the wavefunction in order to incorporate in the calculation of the scattering rate in self-consistent carrier transport model^18^.

The Ben Daniel-Duke model is one of the simplest models for band structure calculation. A more detailed method which intrinsically include the nonparabolicity, the interaction between different bands, and the strain effect is the **k.p** method. High order **k.p** method is capable of accurately evaluating the band structure of the full-Brillouin zone of bulk materials^25^. While for QCLs working in $\Gamma$-valley, lower order models are enough to include the nonparabolicity effect^26–28^. Δ or *L* valley have been reported to affect the QCL performance due to the current leakage to these valleys^27,28^. To consider the Δ and *L* valley, instead of using the high order **k.p**, band structures are usually treated separately by one band model with different effective masses^27,28^. Moreover, in valence band, because the band mixing is much stronger, effective mass method is not a good approximation to describe the in-plane energy dispersion. 6-band **k.p** has been applied for designing a group-IV QCL^29–31^. Such method explicitly includes the spin interaction and the mixing of the heavy hold (HH), light hole (LH) and split-off (SO) band.

#### Schrödinger-Poisson system

Apart from the intrinsic properties of material, self-consistent Schrödinger-Poisson equation is considered to simulate the band bending caused by charge effect under different doping and carrier distribution^32^. Such approach is known as the mean field treatment of the electron-electron interaction (Hartree approximation). The Poisson equation in 1-D system reads:

2 $$\frac{\partial }{\partial z}\left({\epsilon }_{s}\left(z\right)\frac{\partial }{\partial z}\right)\widetilde{V}(z)=\frac{-q}{{\epsilon }_{0}}\left[{N}_{D}(z)-\sum _{i}{n}_{i}^{s}{\left|{\psi }_{i}(z)\right|}^{2}\right]$$

where *q* is the elemental charge. ${N}_{D}$ is the three-dimensional doping density. And ${n}_{i}^{s}$ is the sheet carrier density of state $i$. The self-consistently solved electrostatic potential $\widetilde{V}$ can potentially have a significant impact on the performance of QCL design as the eigenenergy and wavefunction are altered accordingly, which will further influence the carrier transport^33^. Carrier distribution can be obtained by a specific carrier transport model. However, for simplicity, it is usually evaluated by Fermi-Dirac distribution or Boltzmann distribution to avoid the self-consistent iteration on the most time-consuming carrier transport simulation.

### Carrier transport calculation

#### Carrier transport model

Carrier transport calculation permits to understand the electrical and optical behavior in QCL design. It can be conducted by several methods, including rate equation (RE)^22,34–37^, Monte Carlo (MC)^28,30,38–42^, density matrix (DM)^43–45^ and non-equilibrium Green’s function (NEGF)^46–48^ approach.

Among all these models, RE may be the most straightforward and intuitive way to describe the carrier transport. The transition rate between energy states can be given by experimental measurements^49^. In the advanced ab initio way of modeling, the rate equation needs to be solved in a self-consistent manner because the scattering rate depends on the unknow carrier density. In two-period model, the rate equation reads:

3 $$\frac{d{n}_{i}^{s}}{{dt}}=\sum _{j\ne i}{W}_{{ji}}{n}_{j}^{s}-\sum _{j\ne i}{W}_{{ij}}{n}_{i}^{s}+\sum _{{j}^{{\prime} }\ne i}{W}_{{j}^{{\prime} }i}{n}_{{j}^{{\prime} }}^{s}-\sum _{{j}^{{\prime} }\ne i}{W}_{{{ij}}^{{\prime} }}{n}_{i}^{s}+\sum _{j\ne i}{W}_{j{i}^{{\prime} }}{n}_{j}^{s}-\sum _{j\ne i}{W}_{{i}^{{\prime} }j}{n}_{{i}^{{\prime} }}^{s}$$

where $i,j$ denote for the states in the central period, and ${i}^{{\prime} },{j}^{{\prime} }$ are the states in the adjacent period. ${W}_{{ij}}$ is the scattering time from state $i$ to $j$. The first two terms describe the intra-period transport, and the last four terms describe the inter-period transport. Then the equation is solved under steady state after self-consistent iteration reaches convergence. Optical field coupling can be included to describe the carrier dynamics above threshold^50^. Kinetic energy balancing is important in RE method, where the electron temperature is found for zero exceeded kinetic energy, and usually different from lattice temperature^51^.

As modern computer can efficiently generate random number, Monte Carlo method is frequently applied to various complex systems. An improvement of MC method compared to RE method is that it can easily deal with the in-plane carrier dynamics and coupling of the optical field. Different from the RE method, in MC simulation, the carrier transport is simulated by a time dependent dynamics. Within each time subinterval, electrons experience a randomly selected free flight time. The scattering type and final state are further selected randomly according to the ratio of each scattering mechanism. The carrier distribution is then updated after each subinterval. It is worth to mention that numerical approaches should be introduced to minimize the self-scattering time and improve the computational efficiency^38,52^.

Recently, the semiclassical RE and MC method are argued to be inadequate in THz QCL modeling^53–56^. Because in the semi-classical picture, the wavefunction is solved by a well-defined potential profile across the whole structure. The non-meaningful resonance of the wavefunction across a thick barrier near anti-crossing will usually result in an overestimated current density. Thus, density matrix method is adapted which includes the coherent tunneling. The DM is resolved by finding the solution of the von Neumann & Liouville equation. A tight-binding method is usually used. The potential profile is then resolved independently for the local potential (module) separated by a coupling barrier. Thus, the inter-module transition is considered by dephasing and coherent tunneling. The injection barrier is chosen to be the coupling barrier as it usually acts as the ‘bottleneck’ of the carrier transport. However, multiple coupling barrier can also be included within one period to further improve the accuracy of such model^51,53^. The semiclassical RE and MC method are modified based on the DM method in-order to include the dephasing and tunneling effects^22,54–56^. The inter-period scattering rates entering the RE and MC are then replaced by the coherent tunneling rate:

4 $${{\mathcal{R}}}_{{ij}}=\frac{2{\Omega }_{{ij}}^{2}{\tau }_{\parallel ,{ij}}}{1+{\Delta }_{{ij}}^{2}{\tau }_{\parallel ,{ij}}^{2}}{\sigma }_{{ij}}$$

where $${\Omega }_{\begin{array}{c}{ij}\\ \end{array}}$$ is the coupling strength between state *i* and *j*, *τ*_||, *ij*_ is the dephasing time, Δ_*ij*_ is the detuning energy and σ_*ij*_ is the second order current correction which is determined by the detuning energy and carrier distribution. The modified MC and RE is less computational demanding and flexible than DM with compatible accuracy, which makes them efficient tools for the automatic optimization of QCL.

**Fig. 3:**
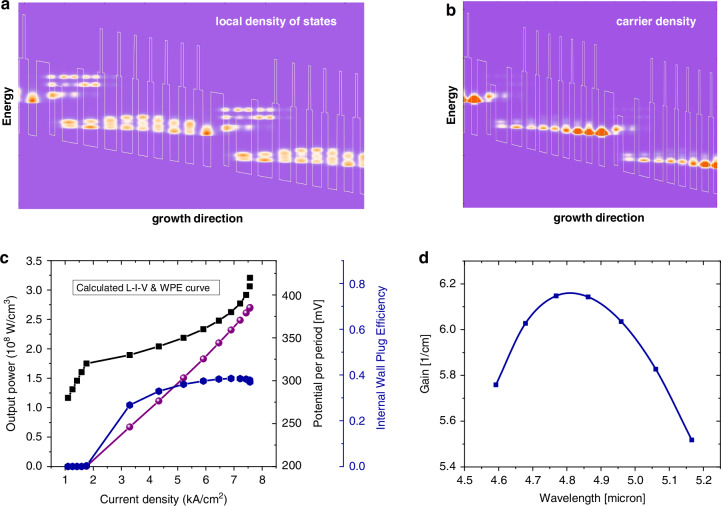
The QCL simulation based on NEGF method. **a** density of states, (**b**) carrier density, (**c**) light-current-voltage (LIV) curves, (**d**) optical gain calculated using NEGF method based on the design in^5^

Lastly, the NEGF is the most general quantum transport model. Both coherent and incoherent transport can be included in such scheme. Different from the previous methods, NEGF does not need the solutions of the Schrödinger equation as input parameters. The transport is described by Dyson and Keldysh equations^47^:

5a $${G}^{R}={(E-{H}_{0}-{\Sigma }^{R})}^{-1}$$

5b $${G}^{ < }={G}^{R}{\Sigma }^{ < }{G}^{R\dagger }$$

5c $${\Sigma }^{ < }={G}^{ < }{D}^{ < }$$

5d $${\Sigma }^{R}={G}^{R}{D}^{R}+{G}^{R}{D}^{ < }+{G}^{ < }{D}^{R}$$

where ${D}^{R}$ and ${D}^{ < }$ are the sum of retarded and lesser Green’s function (${G}^{R}$ and ${G}^{ < }$). ${\Sigma }^{R}$ and ${\Sigma }^{ < }$ are the retarded and lesser self-energies incorporating various scattering mechanisms. With the known Green’s function, the carrier density, current density and optical gain can be well-defined afterwards as shown in Fig. 3. The numerical implementation of NEGF is known to be the most computational expensive. High performance computing (HPC) with supercomputers is usually needed. For such reason, design and optimization of QCL can be conducted by less demanding RE or MC method in order to fast sweep the structural parameters, then the result is further verified by NEGF^57^.

#### Scattering mechanisms

In QCLs, the scattering mechanisms should be included in the carrier transport model. The main scattering mechanisms include phonon scattering^51^, electron-electron scattering^58^, and elastic scattering induced by impurity, interface roughness and alloy disorder^51^. The scattering mechanism is considered by transition rate according to the Fermi’s golden rule while in NEGF the scattering is included in terms of scattering self-energies^47^. Different scattering mechanisms vary between different material systems. For example, in III-V QCLs, polar phonon scattering (i.e. longitudinal optical scattering) is the most important scattering mechanism. Alloy disorder scattering is significant in material system like InGaAs/InAlAs, however in GaAs/AlGaAs THz QCL with pure material in well layer, alloy disorder scattering plays a minor role as most of the carriers are confined in the well region. Besides, screen correction must be included. In coulombic scattering (e-e and impurity), random-phase-approximation is an accurate screen model by solving a set of linear equations of the Coulomb matrix elements. For simplicity, constant inversion screen length derived from Debye and Thomas-Fermi model is usually used. For phonon scattering, Debye’s model can also be used for screening. Additional corrections from hot phonons effect may also be considered^59^.

## How to achieve high wall-plug-efficiency QCLs

The general strategy for QCL wall-plug efficiency (WPE) (*η*_*w*_) optimization is: (1) to optimize WPE in pulsed mode; (2) then try to push the cw WPE as close to the pulsed WPE as possible using efficient thermal dissipation packaging. The pulsed WPE of a QCL can be decomposed into four sub-efficiencies, i.e., the internal quantum efficiency (*η*_*i*_), voltage efficiency (*η*_*v*_), electrical efficiency (*η*_*e*_), and optical efficiency (*η*_*o*_). Then the WPE can be improved by enhancing some of the four sub-efficiencies without sacrificing the others. Otherwise, a compromise among these parameters needs to be made^60^.

WPE is defined as the input electrical power converted into the output optical power:

6 $${\eta }_{w}=\frac{P}{IV}$$

Where *P* is output optical power, *I* is injected current and *V* is voltage applied to a QCL. *P* and *V* in Eq. (1) can be further replaced by Eq. (7) and Eq. (8) as following:

7 $$P={\eta }_{s}(I-{I}_{th})$$

8 $$V={V}_{th}+(I-{I}_{th})R$$

Where *η*_*s*_ is slope efficiency, defined as the increase in output power per unit increase in drive current, *V*_*th*_ is threshold voltage, *I* is current, *I*_*th*_ is threshold current, *R* represents differential resistance, defined as the slope of voltage over current. Then we can factorize the WPE into four components:

9 $${\eta }_{w}={\eta }_{i}{\eta }_{o}{\eta }_{v}{\eta }_{e}$$

with the following expression for the optical efficiency (*η*_*o*_), voltage efficiency (*η*_*v*_), electrical efficiency (*η*_*e*_) and internal quantum efficiency (*η*_*i*_), respectively^60^:

10 $${\eta }_{o}=\frac{{\alpha }_{m}}{{\alpha }_{m}+{\alpha }_{w}}$$

11 $${\eta }_{v}=\frac{N\hslash \omega }{e}\frac{1}{{V}_{th}}$$

12 $${\eta }_{e}=\frac{I-{I}_{th}}{I\left[1+\frac{R(I-{I}_{th})}{{V}_{th}}\right]}$$

13 $${\eta }_{i}={\eta }_{{inj}}-\frac{{\tau }_{l}^{* }}{{\tau }_{u}\left(1-\frac{{\tau }_{l}^{* }}{{\tau }_{{ul}}}\right)+{\tau }_{l}^{* }}$$

In above formulas, *α*_*m*_ is mirror loss, *α*_*w*_ is waveguide loss, *N* is number of QCL stage, *ħω* is photon energy of QCL emission, *η*_inj_ is the injection efficiency of QCL, defined as the ratio between the current density injected to the upper laser level to the total current density, which is a reflection of leakage current to other levels, *τ*_u_ is upper laser level lifetime, *τ*_l_ is lower laser level lifetime, *τ*_ul_ is transition time between upper and lower laser level.

In Eqs. (9–13), each sub-efficiency is dimensionless but physically meaningful. Additionally, the current-dependent nature of WPE is only included in the electrical efficiency. The *η*_*e*_ can be reformed to be in terms of the current density (*J*) and threshold current density (*J*_*th*_)^60^:

14 $${\eta }_{e}=\frac{J-{J}_{th}}{J\left[1+\frac{RA(J-{J}_{th})}{{V}_{th}}\right]}$$

**Fig. 4:**
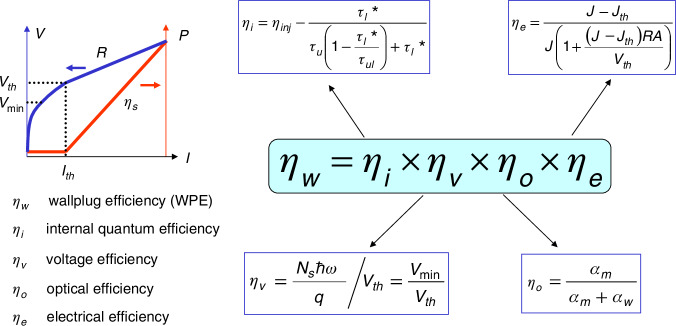
The components and the physical meaning of these parameters that related to QCL WPE. *α*_*m*_ mirror loss, *α*_*w*_ waveguide loss, *N*_*s*_ number of QCL stage, *ħω* photon energy of QCL emission, *V*_*th*_ threshold voltage, *I* current, *I*_*th*_ threshold current, *R* differential resistance, *A* device area, *J* current density, *J*_*th*_ threshold current density, *τ*_u_ upper laser level lifetime, *τ*_l_ lower laser level lifetime, *τ*_ul_ transition time between upper and lower laser level. The differential resistance R is evaluated just above threshold, where the laser transitions into the stimulated emission regime

Where the product of RA is differential resistance R multiplied by the cross-section area (A) of the current and the differential resistance R is evaluated just above threshold, where the laser transitions into the stimulated emission regime. The WPE decomposition process is summarized in Fig. 4. Improve WPE of QCL is arguably one of the most challenging tasks in QCL community as it is strongly related to all device parameters as mentioned above and in Fig. 4 as well. We need to make clear their underlying relationships and establish solid connection between changes made to the structure and their consequences to the device performance parameters.

The internal quantum efficiency (*η*_*i*_) represents the intrinsic properties of QCL wafer. It is mainly determined by the injection efficiency and the lifetime of electrons on upper and lower energy levels as given in Fig. 4. This intrinsic parameter is believed to be the most important figure of metric for evaluating the quality of wafer^61,62^. Actually, we can accurately obtain this parameter through mirror loss dependent slope efficiency (*η*_*s*_) measurement. However, due to the complexity of the QCL structure, theoretical prediction of internal quantum efficiency is usually based on different approximations, which may lead to unignorable discrepancy.

The voltage efficiency is defined as the ratio between the minimum voltage to the threshold voltage as given in Eq. (11) or in Fig. 4. The minimum voltage ${V}_{\min }=\frac{N\hslash \omega }{e}$ is only related to the lasing frequency and the total number of QCL periods. The threshold voltage is always higher than the minimum voltage due to the injector resistance (internal) in laser core and parasitic contributions from cladding layer, top layer and the semiconductor-metal contact (external).

The electrical efficiency given in Eq. (12) is the only sub-efficiency depending on current density. Letting $\frac{d{\eta }_{e}}{dJ}=0$, we have:

15 $$J\left[1+\frac{RA(J-{J}_{th})}{{V}_{th}}\right]-(J-{J}_{th})\left\{\left[1+\frac{RA(J-{J}_{th})}{{V}_{th}}\right]+J\frac{RA}{{V}_{th}}\right\}=0$$

After simplification, we have the following equation:

16 $$\frac{RA{(J-{J}_{th})}^{2}}{{V}_{th}}-{J}_{th}=0$$

Then we get the solutions to Eq. (15):

17 $$J={J}_{th}\pm \sqrt{\frac{{J}_{th}{V}_{th}}{RA}}$$

We forsake the smaller solution due to the physical constrain of ${J}_{peak} > {J}_{th}$. Then we have the following peak current density solution:

18 $${J}_{peak}={J}_{th}+\sqrt{\frac{{J}_{th}{V}_{th}}{RA}}$$

Finally, ${{\eta }_{e}}^{\max }$ can be obtained by substituting $J={J}_{peak}$ into ${\eta }_{e}(J)$, resulting in:

19 $${{\eta }_{e}}^{\max }=\frac{1}{{\left(\sqrt{\frac{{J}_{th}RA}{{V}_{th}}}+1\right)}^{2}}$$

The optical efficiency *η*_*o*_ is the ratio of the mirror loss to the total loss as given in Eq. (10). The mirror loss is related to cavity length and facet reflectivity. The waveguide loss can be obtained from the mirror loss dependent slope efficiency measurement^12^. From Eq. (10), the optical efficiency increases with the increase of mirror loss and the decrease of waveguide loss. However, increasing the mirror loss will result in a higher threshold current density, which will decrease the electrical efficiency and the voltage efficiency. Actually, there exists an optimized mirror loss, making WPE reach its maximum. The analytical solution to achieve this optimum condition is complicated. As mirror loss is related to cavity length through: α_m_ = (1/L)·log{1/(R_1_·R_2_)^1/2^}, where L is the cavity length, R1, R2 are the facet reflectivity, we practically plot the current density dependent WPE for different cavity length. Then the cavity length that gives the highest WPE can be obtained.

## Mid-Infrared QCL

For QCLs, there are two mainstream categories of material system, one is GaAs-based material system and the other is InP-based material system. The GaAs-based GaAs/AlGaAs material system is generally used to realize THz QCLs and the InP-based InGaAs/AlInAs material system is used for mid-infrared QCLs. After approximately 30 years’ efforts, the performance of mid-infrared QCLs has been considerably increased in operating temperature^8,63^, output power^5,7^, WPE^5,64^, brightness^65^ etc. As one of the first groups who have the full capability from QCL band structure design, active material growth, and device fabrication, testing and packaging, the CQD has made a plethora of pivotal contributions to the development of QCLs. Some of the achievements are listed in detail^8,63,66–71^. In this part, we will review the CQD recent progress of our research on high-brightness, high-power, high WPE mid-infrared QCL development in CW operation at room temperature.

### High-power mid-infrared QCL in CW operation

**Fig. 5:**
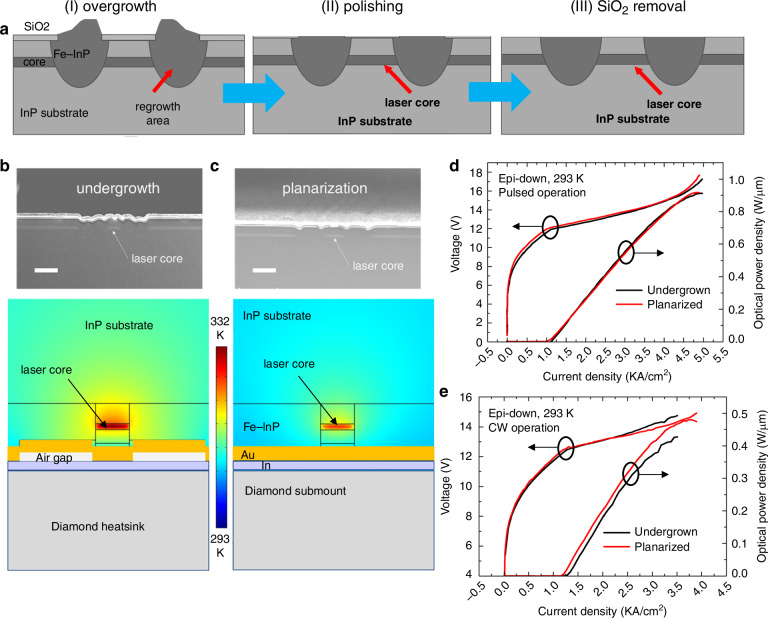
The regrowth planarization technique of QCLs. **a** Schematic of the processing flow for the redesigned buried ridge process. **b** The SEM image of an epilayer-down bonded undergrown QCL device (upper panel) and the device thermal distribution simulated with the finite-element method (lower panel). **c** The SEM image of an epi-down bonded planarized QCL device using the redesigned processing technique (upper panel) and the thermal simulation result of the device (lower panel). The two simulation figures share the same scalar bar to reflect the internal temperature difference for the two cases. The white bars on the SEM images represent a length scale of 10 μm. **d** *P*–*J*–*V* characterizations for the undergrown and planarized QCLs in pulsed mode operation (**d**) and in CW operation (**e**)^7^

In part 2, we have discussed the methodology to increase the WPE of QCLs. Actually, increasing WPE is the root strategy and first step for increasing the overall cw performance of QCLs. This sub-part, we will discussion how to improve the output power of QCLs in cw operation at room temperature after pulsed WPE optimization. To improve the cw performance of QCLs, optimizing the active region is generally the priority choice, which includes the optimization of the quantum efficiency, characteristic temperature, lasing threshold etc. However, this kind of optimization involves many rounds of band structure design, active material growth, device fabrication, testing and packaging, consuming much energy, money and time. Another approach is to optimize device fabrication process. In semiconductor laser fabrication, laser ridge needs to be etched out from a flat wafer. These etched channels will considerably decrease thermal extraction efficiency from laser core, preventing high-power CW laser operation. The buried ridge regrowth process^4,72^ is developed to solve above problem and now has been widely used in the fabrication of high-power CW QCLs^12^. It uses metalorganic chemical vapor deposition (MOCVD) method to fill the etched channels around laser cores and thus increase the thermal disspation of QCLs to achieve high-power CW operation. However, one shortcoming of this standard process is that the regrowth area by Fe-doped InP is not flat as the regrowth rate depends on many factors, including the opening channel shape, volume of wet etching channels, density of the channel patterns etc. These wavy areas as shown in Fig. 5a can potentially introduce air gaps during device epi-layer down bonding on heat sink, resulting in low heat dissipation efficiency or even burn the devices in cw operation.

In our recent effort, we tried to improve the cw performance of QCL devices by bringing in a post-polishing technique after buried ridge regrowth process for achieving better surface planarization^7^, which is capable of improving the device reliability, thermal dissipation efficiency and cw performance. The schematic of the processing flow for the new buried ridge process was given in Fig. 5a. The detailed description of this process can be found in^7^. Figure 5b, c show the scanning electron microscope (SEM) images (upper panels) and the temperature distribution (lower panels) of an undergrown device and a planarized device of a QCL device driven to be near the lasing threshold in CW operation, from which we can see that the epilayer-down bonded, planarized device exhibited a lower internal temperature (5 °C lower), leading to an improved reliability and thermal performance compared with the undergrown ones.

**Fig. 6:**
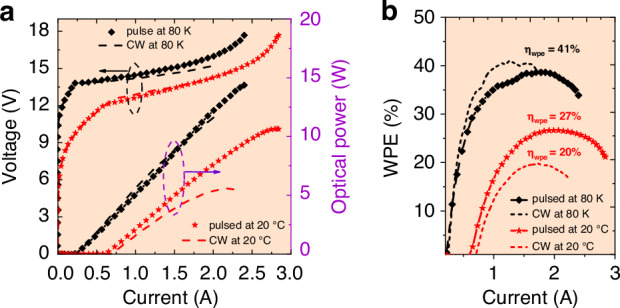
The characteristics of high performance QCL based on the regrowth planarization technique. **a** The LIV curves of the QCL working at 80 K (black) and 20 °C (red) in pulsed (dashed) and cw (solid) modes, respectively. Inset: the lasing spectra of the QCL at a driving current of ∼0.8 A at room temperature (red) and at a current of ∼0.5 A at liquid nitrogen temperature (black). **b** The WPE of the QCL in (**a**)

Figure 5d, e shows the power - current density - voltage (P–J–V) characterizations of these two QCLs in pulsed mode and CW operation, respectively. In pulsed mode operation, their P–J curves were almost overlapped. However, in cw operation the planarized device shows a lower threshold density dropping from 1.22 kA/cm^2^ to 1.16 kA/cm^2^ and an improved maximum output power density from 0.42 W/μm to 0.48 W/μm, owing to the increased thermal dissipation efficiency of the planarized device compared with the undergrown device. Based on this regrowth planarization technique, high-power high-WPE QCLs have been achieved in cw operation. As shown in Fig. 6a, b, the CW-WPE reached 41% at 80 K and reached 20% at 20 °C. The cw output power from a single anti-reflection (AR) coated facet has exceeded 12 W at 80 K and exceeded 5.6 W at 20 °C.

### High Wall-plug efficiency QCLs at CQD/NU^5^

Unlike traditional semiconductor lasers whose efficiency is capable of exceeding ∼70% at room temperature^73^, QCLs are intrinsically high energy consuming semiconductor devices as a minimum voltage (typically above 10 V) is required to align the cascade structures and a high threshold current density (typically 1 kA/cm^2^) is needed to compensate the total loss before exhibiting any lasing behaviour^5,64^. Improving the global efficiency of QCLs can not only reduce the electrical energy consumption, but also can minimize the waste heat produced within a laser and significantly improve the reliability of lasers, especially for QCLs in CW operation. Today, saving energy and mitigating global warming are one of the most important themes of our world. Thus, developing high efficiency devices or equipment is a main goal in many different areas.

As presented in part 2, the WPE of a QCL represents the energy conversion efficiency from electrical power input to optical power output. It is a direct indicator of the energy usage efficiency of lasers. In our recent work, we have successfully increased the WPE of QCLs in both pulsed mode and CW operation by optimizing the thickness of laser core without modifying its band structure^5,64^.

As we know, the pulsed WPE of QCLs can be easily augmented by increasing the number of QCL stages, *Ns*, since it can increase the waveguide optical confinement factor (Г), reduce the waveguide loss (*α*_*w*_), and decrease the threshold current density (*J*_*th*_). However, simply increasing *Ns* will directly lead to much stronger internal heating and therefore, result in sharp performance degradation of QCL devices when working in CW condition. So, in order to get high CW WPE, an optimization of the thickness is required by taking into account the optical and thermal consequences. This is the way that we improved the CW WPE of QCLs^5^.

**Fig. 7:**
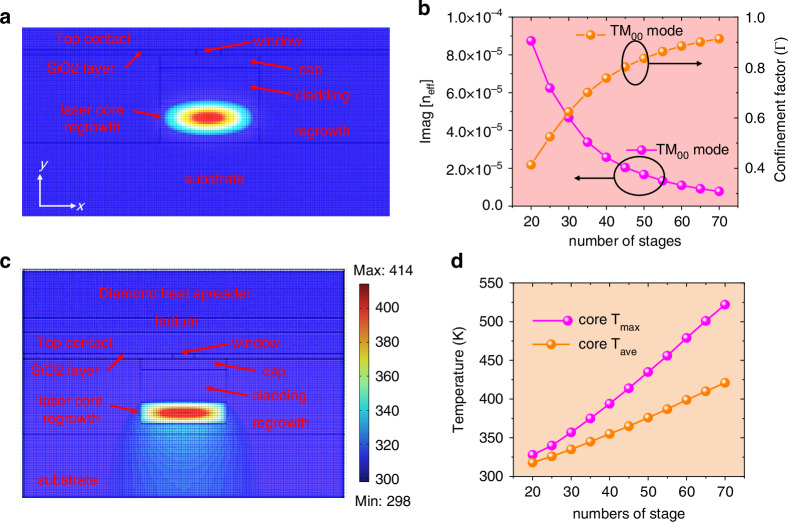
The optical and thermal simulations of a QCL. **a** The optical modal simulation of a buried ridge QCL. **b** The imaginary part of the effective refractive index (proportional to waveguide loss) and the confinement factor of the buried QCL as a function of the numbers of QCL stages. **c** The model and temperature distribution of a buried ridge QCL epi-down bonded on a diamond heat spreader for CW operation. **d** The maximum (*T*_*max*_) and average (*T*_*average*_) core temperature of the buried ridge QCL under CW operation as a function of QCL stages

Figure 7 gives the optical simulation and thermal simulation of a QCL and shows us how the optical property and thermal property vary as a function of QCL stage number. Detailly, Fig. 7a shows the optical simulation model of a buried ridge QCL approximating to our real device and (b) shows the waveguide loss and the confinement factor of the buried ridge QCL as a function of the numbers of QCL stages. Figure 7c shows the thermal simulation model and temperature distribution of the buried ridge QCL epi-layer down bonded onto a diamond heatsink. Figure 7d shows the maximum (T_max_) and average (T_average_) temperature of the buried ridge QCL core under CW operation as a function of the number of QCL stage. According to the optical and thermal calculation, increasing the QCL stage from 40 to 45 can potentially increase the pulsed WPE from 27% to 29% and increase the CW WPE from 21% to 22%^5^.

**Fig. 8:**
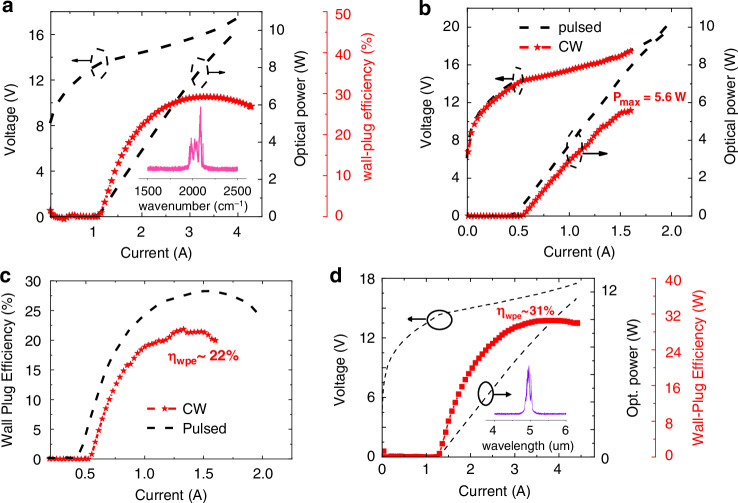
The experitental results of the optically and thermally optimized QCL. **a** The LIV curves and extracted WPE for a 5 mm long, 17 μm wide laser working in pulsed mode. Inset: the spectrum of the laser emission at 2.5 kA/cm^2^. **b** The LIV curves and (**c**) extracted WPE of the HRAR coated, buried ridge QCL with 8-μm ridge width and 5 mm cavity length in CW operation at 20 °C. **d** The LIV curve and the WPE of the buried laser with 50 QCL-stages as a function of current. Inset: the spectrum of the laser emission at 1.6 A. This testing was conducted in pulsed mode with a pulse width of 500 ns and a duty cycle of 2%

Figure 8 shows the experimental results of this optimization. In pulsed operation at room temperature, the output power was over 10 W and its WPE was 29.3%, very close the predicted value. The internal quantum efficiency, waveguide loss, transparency current density, and modal gain of this laser were measured to be 76%, 0.37 cm^−^^1^, 0.6 kA/cm^2^, and 5 cm/kA, respectively. In CW operation, the output power of the laser from one single facet at room temperature was above 5.6 W, breaking the record of the MWIR spectral range by 10%^5^. The CW WPE was measured to be ~22%, also very close to the predicted value and representing the new record of QCL efficiency up-to-date in the whole mid-infrared and THz spectral range. We also further increased the QCL stage number to 50 as shown in Fig. 8c. The pulsed WPE was further increased to be 31% at room temperature and an output power was measured to be approximately 12W^64^. These results are WORLD record for highest power, highest WPE in pule and CW operation.

It worth mention that A. Lyakh et al. reported a QCL with pulsed WPE exceeding 28%^74^. But this QCL did not succeed in achieving high WPE in CW operation at room temperature due to its relatively low characteristic temperature T_0_ ~ 140 K.

### High brightness LWIR QCLs at CQD/NU^65^

Long-wave infrared (LWIR) QCLs are drawing more and more attentions in the infrared domain as they are located at the atmosphere telecommunication window and capable of providing long-distance transmission of infrared light in free space^75–77^. However, the development of LWIR QCLs has been lagging behind the shorter wavelength QCLs due to increased free-carrier absorption, lower inter-sub-band gain coefficient etc. If using thicker active layers to compensate waveguide loss increase and optical confinement factor decrease, it will lead to stronger internal heating and worse thermal performance.

Scientists at CQD adopted a highly localized diagonal laser transition strategy to tailor the band structure and designed an out-coupler based on an electrically isolated taper structure for light extraction upscaling of LWIR QCLs^65^. Based on this solution, we demonstrated high beam quality single-mode QCLs spanning from λ ≈ 8–10 μm with high cw brightness light extraction from a single facet at 15 °C.

**Fig. 9:**
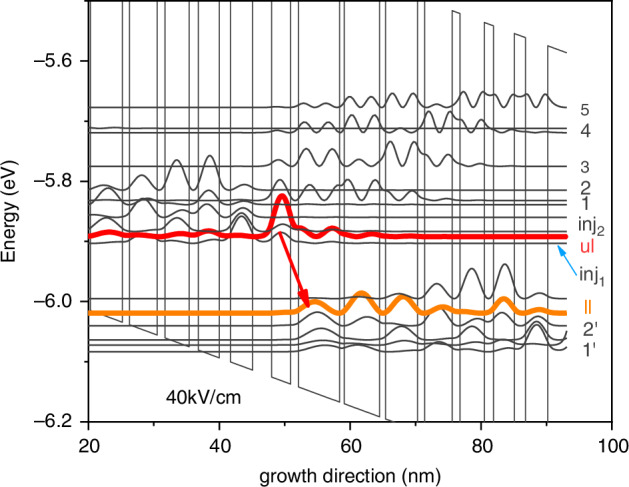
Engineered band structure of LWIR QCL with an emitting wavelength at λ ≈ 10 μm

Figure 9 shows the band structure of the QCL. In this design, the laser was engineered to be diagonal transition for having a longer upper-level lifetime. The energy difference between the two laser transition levels was about 126 meV, corresponding to an emitting wavelength at λ ≈ 10 μm. The wave function of the upper transition level was engineered to be highly localized in the active region, which could prevent the back transport and forward spreading of electrons into the injector areas and help electrons efficiently accumulate in the active region.

The wave function of the lower energy level was tailored to be non-localized within the active area, which provided an additional channel for electron transport from the active area to the injector region beside the optical phonon extraction. Beside the internal limitation such as the increased free-carrier absorption, lower inter-sub-band gain coefficient, one key external limitation for realizing high-brightness QCLs in the LWIR spectral range is the high-power extraction from the laser cavity. Unlike MWIR QCLs whose power can be easily extracted through facet coating, the LWIR QCLs will bring a strong optical absorption on the coated facet, especially for the wavelengths over 10 μm. In addition, a much thicker (~2 times) coating layer required for LWIR QCLs further enhances the optical absorption of output facet. This strong facet heating can considerably decrease the output power and damage the output facet. This is why all the reported QCLs with emitting wavelengths around λ ≈ 10 μm were always uncoated, and the claimed high power was actually the total power from two facets. Scientist demonstrated that yttrium oxide is one of the best materials for high-brightness LWIR QCL coating. Even so, its output facet can be easily damaged when the output light brightness is nearly 0.7 MWcm^−^^2^sr^−^^1^ in cw operation.

**Fig. 10:**
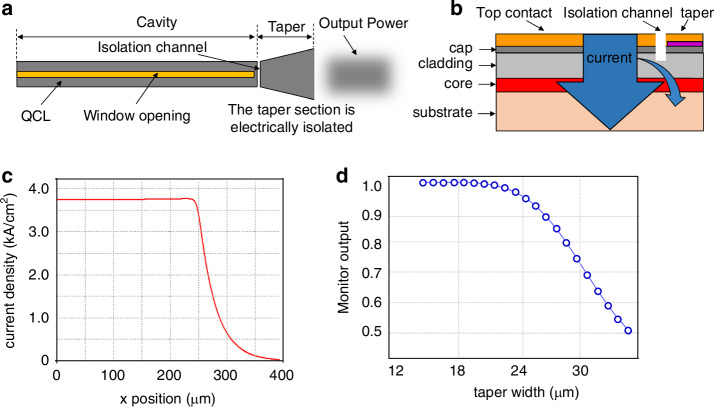
Scheme for the facet damage of long-wave mid-infrared QCL. **a** Schematic of a QCL with an electrically isolated tapered output structure. **b** Cross-sectional view of the QCL using the electrically isolated taper structure scheme. **c** Current spreading into the tapered out-coupler along the laser cavity direction with an isolation channel depth of 2 um. **d** Output power of the fundamental mode (TM_00_) as a function of the taper width on the output facet^65^

To solve this problem, Razeghi’s group designed an out-coupler using an electrically isolated taper structure as shown in Fig. 10a. This coupler was able to extend the laser beam and decrease the output optical density, but would not bring any additional electrical heating to the facet in the LWIR ranges. Figure 10b shows the geometry structure of the QCL using the electrically isolated taper structure and Fig. 10c shows the current density spreading into the tapered out-coupler along the laser cavity direction with an isolation channel depth of 2 µm. For a 100 μm long taper, the current density near the output facet is significantly reduced to be only 5% of the current density on the laser part, resulting in ignorable electrical heating^78^. Figure 10d shows the optical calculation of this tapered QCL waveguide. The aim was to decide the geometry parameters of the taper for keeping fundamental transverse mode (TM_00_) propagation and high beam quality for practical use.

**Fig. 11:**
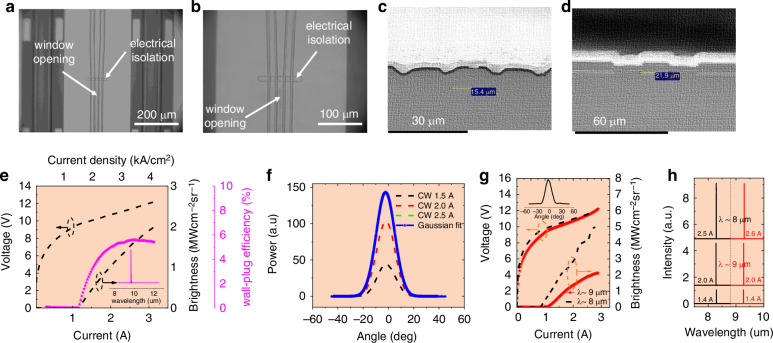
The optical damage mitigrated long-wave mid-infrared QCL. Optical images of the interface between the taper and the QCL device with (**a**) lower and (**b**) higher magnification. **c** SEM image of the back facet of the buried ridge QCL device. **d** SEM image of the taper side facet of the buried ridge QCL device. **e** LIV-WE curves of the QCL with a tapered structure. Inset: the spectrum of the laser emission at 3 A. **f** Far field of the QCL at different current injections in CW operation. **g** LIV curves of the tapered QCLs with DFB gratings targeted at λ ≈ 8 and 9 μm, respectively. Inset: the far field of the QCL at λ ≈ 8 μm with an injection current of 1.8 A. **h** Spectra of the two lasers as a function of current from 1.4 to 2.6 A^65^

Figure 11a, b shows the optical images of the interface between the taper and the QCL device. Figure 11c, d shows the SEM images of the back and front facet of the real QCL devices. The lower panel of Fig. 11 shows the LIV characteristics, far-field, and spectra of this kind of lasers with an emitting wavelength at λ ≈ 8–10 μm. The brightness of the QCL at λ ≈ 10 μm was considerably increased to be over 2 MWcm^−2^sr^−1^, breaking the record by approximately 2 times^79–82^. In the same work, the brightness of QCL with DFB grating targeted at λ ≈ 8 μm and 9 μm was also increased to be more than 5 MWcm^−2^sr^−1^ and 2.2 MWcm^−2^sr^−1^, respectively, both of which are current record.

## THZ QCL

### High-power High-temperature THz QCLs

THz QCLs typically operate at low temperatures to achieve high output power. However, recent research has significantly increased the working temperature of QCLs by optimizing active region design, the material component, growth technique etc. This has led to some QCL devices that can operate at temperatures as high as 261 K^10^. This is of great significance for the practicality and wide application of QCLs in diverse applications.

When the first THz-QCL was born in 2002, its maximum operating temperature was around 50 K^13^. Three years later, Hu’s group from MIT increased the operating temperature to 164 K^15^. Their strategy was to efficiently extract electrons from the lower-energy level using a resonant phonon scheme to make population inversion easier to be achieved, and to increase the optical confinement factor (Γ) from 0.1 − 0.5 to 1 using a metal-metal (MM) waveguide. Although MM waveguide loss is larger, the operating temperature of THz-QCL was significantly improved. A few years later, Hu’s Group collaborated with researchers from University of Waterloo further increased the temperature to about 200 K by optimizing the coupling strength between transition energy levels and improving injection efficiency of electron tunneling^83^.

In 2019, Faist’s Group systematically optimized the two-quantum well active structure using NEGF method and increased the operating temperature to 210 K^11^. Two years later, Hu’s group increased it to 250 K by adopting a two-well three-level system design and increasing the *Al* component to 30%^9^. Recently, the operating temperature of THz QCLs was further increased to 261 K by the same Group from MIT through further optimizing previous result^10^.

Despite considerable progress has been achieved on the operation temperature of QCLs, it remains a big challenge for the scientists to realize room temperature THz QCLs. It is worth noting that an approach based on difference frequency generation in the intracavity between a two-color mid-IR QCL has recently allowed THz emission at room temperature in a monolithic mid-IR QCL device^84,85^. This idea was first introduced by Belkin et al. ^86^. Then Razeghi’s Group improved the performance of mid-IR QCLs and led to the room-temperature high-power performance of such devices^84,85^. Besides, the THz output has also reached 2.4 W at 10 K and 1.8 W at 77 K on a single, 24 μm-thick active region QCL with single-plasmon THz waveguide by researchers from Leeds University^6^.

## Summary And Discussion

The field of QCLs has witnessed remarkable advancements in the past years, yet the quest for room temperature high-power cw operation in THz regime remains an ongoing challenge. However, based on the progress made so far, as well as emerging technologies and research directions, there are promising avenues to explore in order to achieve room temperature high-power THz semiconductor lasers diodes and higher WPE QCLs. This section discusses potential strategies and future prospects for advancing THz laser technology.

### THz Quantum Cascade Lasers

Modelocked THz QCLs can be widely used in both science and industrial applications in the THz spectral regime as diverse as non-linear optics, ultrafast physical process investigation, spectroscopy and more recently optical frequency combs. Unlike traditional semiconductor lasers, modelocking of QCL has proven to be extremely challenge due to its ever-ultrafast gain recovery time in picosecond timescale^87–91^.

**Fig. 12:**
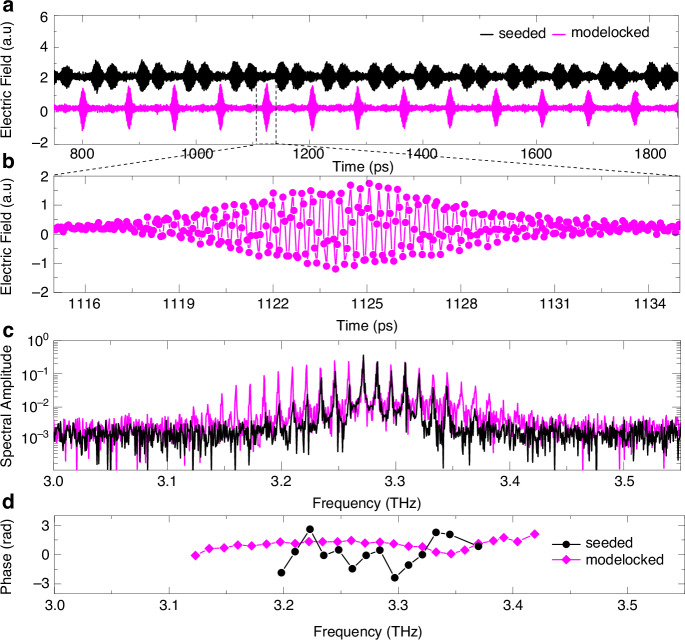
Free running and mode-locked emission of a THz QCL at 3.25 THz, which were measured using injection seeding technique. **a** Time resolved electric field of a free-running (black) and mode-locked (magenta curves) QCL emission from 750 ps to 1850 ps. **b** The zoom in of the mode-locked pulse at 1125 ps in (**a**). **c** Spectra of the free-running and mode-locked emission of the QCL through Fourier Transform of the time-resolved electric field in (**a**). **d** Phase of each lasing mode shown in (**c**) under free-running and mode-locked conditions, respectively

However, THz QCL mode-locking has been demonstrated for the first time in 2011 through modulating the bias current^92^. In their scheme, detection of the emitted pulse train was made possible by phase-locking the QCL repetition rate and carrier frequency to a harmonic of the repetition rate of a mode-locked femtosecond fiber laser. This technique allows control of the carrier-envelope phase shift of the QCLs, but not able to direct observe the pulse or measure the pulse width. One year later, QCL modelocking was demonstrated in time domain using the “injection seeding” technique^93–95^. This approach provides the possibility to directly sampling the electric field of THz QCL as shown in Fig. 12, in which (a) is the electric field of a QCL emission from 750 ps to 1850 ps. 12 (b) shows the zoom in of the mode-locked pulse at 1125 ps given in 12 (a). 12 (c) shows the QCL spectra obtained through Fourier Transform of the time-resolved electric field in 12 (a). 12 (d) shows the phase of each lasing mode shown in 12 (c) under free-running and mode-locked conditions, respectively.

In 2015, researchers from École Normale Supérierue found that contrary to a long-standing belief that the QCL gain dynamics are the limiting factor, the key mechanism is actually a nonlinear interaction between the generated pulse and the applied electrical modulation^87^. This important information has permitted new avenues to be explored to generate shorter and intense THz pulses from a QCL.

To further compress the pulse width of mode-locked THz QCLs, a monolithic on-chip dispersion compensation scheme was proposed^96^ by the Group at École Normale Supérierue. This was based on the realization of a small coupled cavity resonator that acted as an ‘off resonance’ Gires-Tournois interferometer (GTI), permitting large THz spectral band-widths to be compensated^96^. In this work, the THz pulses from mode-locked QCLs was considerably compressed from 16 ps to 4 ps. This result marks an important milestone in exploring ultrafast light pulse generation from mode-locked QCLs. The novel application of a GTI also opens up a route to sub-picosecond and single cycle pulses in the THz range from a compact semiconductor source. Based on these techniques and experimental results, the ultrafast dynamics of mode-locked and tuneable THz QCLs were also investigated^97–99^. This demonstration marks an important formulism for future progress towards exploring the ultrafast pulse generation buildup dynamics of these complex semiconductor lasers^97–99^.

Recently, researchers were also attempting to explore the mechanisms and experiments of passive mode-locking of THz QCL, and some progresses have been achieved^100–102^. In addition, QCL mode-locking has also been extended to mid-infrared regime^103,104^. Short pulses in femtosecond timescale have been obtained using additional pulse compression techniques^104^.

QCLs have demonstrated great potential for THz emission. Besides ultrafast THz QCLs, future efforts should focus on refining the design and engineering of QCLs to achieve higher output powers at room temperature. This includes optimizing the active region, injector design, and waveguide configurations, as well as incorporating advanced cavity designs and novel gain media.

### Mid-infrared Quantum Cascade Lasers through DFG

Improving THz power emission through DFG in mid-infrared QCLs requires careful optimization of various aspects of the device. First, it needs to use numerical modeling tools to optimize the DFG process and guide device design. Numerical simulations can provide insights into waveguide designs, nonlinear material properties, phase matching conditions, and optimize the DFG efficiency and THz power emission. By optimizing the active region design, nonlinearity of materials, the waveguide structure, achieving phase matching, enhancing output power, optimizing cavity design and feedback, implementing efficient cooling techniques, and utilizing numerical modeling can help us further improve the THz power emission.

### Material innovations

Continued research into advanced semiconductor materials is key to realizing room temperature high-power THz lasers diodes. Exploring new material compositions (such as SiGeSn, GaN), heterostructures, and alloys can offer enhanced energy levels, barrier potential, carrier transport properties, and reduced scattering losses provide possibilities for THz laser diodes development.

### Hybrid laser architectures

Exploring hybrid laser architectures that combine multiple laser sources can provide a path towards high-power THz lasers. Integrating THz QCLs with other laser technologies, such as semiconductor disk lasers or solid-state lasers, can offer power scaling and spectral coverage advantages. Investigating new integration schemes and optimizing coupling techniques will be instrumental in achieving high-power THz emission.

### Power scaling and beam control

Developing strategies to scale the output power of THz lasers is vital for practical applications. Advancements in array technologies, phase-locked laser systems, and beam combining techniques can enable significant power scaling and improved beam quality. Additionally, the exploration of beam shaping and control methods, including adaptive optics and metasurface-based devices, can enhance the spatial and spectral characteristics of THz laser beams.

In conclusion, while the realization of room temperature high-power semiconductor THz laser diodes and higher WPE mid-IR QCLs poses significant challenges, the future prospects are promising. Continued research and development in material science, active region design, hybrid architectures and power scaling strategies will pave the way for achieving room temperature high-power THz laser diodes and higher WPE QCLs. These advancements hold immense potential for enabling a wide range of applications in areas such as spectroscopy, imaging, communication, and sensing, thus unlocking new opportunities in IR and THz science and technology.

## Data Availability

The data that support the plots within this paper and another finding of this study are available from the corresponding author upon reasonable request.
